# Cross-species transcriptome analysis for early detection and specific therapeutic targeting of human lupus nephritis

**DOI:** 10.1136/annrheumdis-2021-222069

**Published:** 2022-07-29

**Authors:** Eleni Frangou, Panagiotis Garantziotis, Maria Grigoriou, Aggelos Banos, Dionysis Nikolopoulos, Antigone Pieta, Stavros A Doumas, Antonis Fanouriakis, Aikaterini Hatzioannou, Theodora Manolakou, Themis Alissafi, Panayotis Verginis, Emmanouil Athanasiadis, Emmanouil Dermitzakis, George Bertsias, Anastasia Filia, Dimitrios T Boumpas

**Affiliations:** 1 Laboratory of Autoimmunity and Inflammation, Biomedical Research Foundation of the Academy of Athens, Athens, Greece; 2 Department of Nephrology, Limassol General Hospital, Limassol, Cyprus; 3 Department of Clinical Immunology and Rheumatology, Medical University Hannover, Hannover, Germany; 4 Department of Genetic Medicine and Development and Institute of Genetics and Genomics of Geneva (iG3), University of Geneva Medical School, Geneve, Switzerland; 5 Laboratory of Rheumatology, Autoimmunity and Inflammation, University of Crete Medical School, Heraklion, Greece; 6 4th Department of Medicine, National and Kapodistrian University of Athens Medical School, Athens, Greece; 7 Medical School, University of Cyprus, Nicosia, Cyprus

**Keywords:** Systemic Lupus Erythematosus, Lupus Nephritis, Therapeutics, Autoimmunity, Autoimmune Diseases

## Abstract

**Objectives:**

Patients with lupus nephritis (LN) are in urgent need for early diagnosis and therapeutic interventions targeting aberrant molecular pathways enriched in affected kidneys.

**Methods:**

We used mRNA-sequencing in effector (spleen) and target (kidneys, brain) tissues from lupus and control mice at sequential time points, and in the blood from 367 individuals (261 systemic lupus erythematosus (SLE) patients and 106 healthy individuals). Comparative cross-tissue and cross-species analyses were performed. The human dataset was split into training and validation sets and machine learning was applied to build LN predictive models.

**Results:**

In murine SLE, we defined a kidney-specific molecular signature, as well as a molecular signature that underlies transition from preclinical to overt disease and encompasses pathways linked to metabolism, innate immune system and neutrophil degranulation. The murine kidney transcriptome partially mirrors the blood transcriptome of patients with LN with 11 key transcription factors regulating the cross-species active LN molecular signature. Integrated protein-to-protein interaction and drug prediction analyses identified the kinases TRRAP, AKT2, CDK16 and SCYL1 as putative targets of these factors and capable of reversing the LN signature. Using murine kidney-specific genes as disease predictors and machine-learning training of the human RNA-sequencing dataset, we developed and validated a peripheral blood-based algorithm that discriminates LN patients from normal individuals (based on 18 genes) and non-LN SLE patients (based on 20 genes) with excellent sensitivity and specificity (area under the curve range from 0.80 to 0.99).

**Conclusions:**

Machine-learning analysis of a large whole blood RNA-sequencing dataset of SLE patients using human orthologs of mouse kidney-specific genes can be used for early, non-invasive diagnosis and therapeutic targeting of LN. The kidney-specific gene predictors may facilitate prevention and early intervention trials.

**WHAT IS ALREADY KNOWN ON THIS TOPIC**

- Prediction of patients with systemic lupus erythematosus (SLE) that will develop nephritis and early diagnosis represents an unmet need because of the limited value of known predictors and the invasiveness of kidney biopsy.
- Even with best treatment up to 40% of patients fail to reach a complete renal response suggesting that early diagnosis and prompt treatment including targeting of renal specific pathways is needed.

**WHAT THIS STUDY ADDS**

- Distinct, renal-specific molecular pathways are associated with the development of nephritis and its progression from subclinical to full blown disease in murine SLE.
- The mouse kidney transcriptome mirrors the human whole-blood transcriptome in lupus nephritis (LN).
- Upstream and downstream regulators of the cross-species (murine and human) kidney-specific gene signatures have been identified as putative targets in LN and novel cross-species drug signatures for kidney disease in lupus.
- Using the mouse kidney-specific transcriptome and through training by machine-learning techniques of a large whole-blood RNA-sequencing dataset of SLE patients, we developed and validated an algorithm that predicts patients that will develop LN based on a small number (no more than 20) of genes.

**HOW THIS STUDY MIGHT AFFECT RESEARCH, PRACTICE AND/OR POLICY**

- Common cross-species (murine and human) genes could be prioritised as potential therapeutic targets for LN or tested as an alternative, non-invasive ‘liquid biopsy’ marker of kidney disease in patients with SLE.
- The mouse kidney-specific set of gene predictors may be used towards monitoring human kidney disease in SLE patients and enrolment in LN prevention and early treatment studies.

## Introduction

In lupus nephritis (LN), current therapy fails to induce remission in more than 50% of patients. Even in cases with clinical remission, repeat kidney biopsies often exhibit residual inflammation and increased fibrosis, with 15%–20% of patients eventually developing end-stage kidney disease.^1–3^ Importantly, several clinical trials have failed to meet their primary endpoint^4 5^ with only two new treatments approved for LN.^6–9^ Accordingly, there is urgent need for therapeutic interventions targeting aberrant molecular pathways enriched within the kidneys, to maximise drug efficacy.

Subclinical (silent) LN represents an early stage in the natural history of the disease^10–12^ prior to full-blown disease.^13 14^ Notably, genetic and immunological interventions in lupus models have underscored the potential to avert autoantibody deposition and ensuing immune responses within the kidneys,^15–19^ suggesting that preemptive therapy might represent a valid therapeutic concept.^15 19^ However, the mechanisms underlying the progression to clinical LN are not clearly understood and kidney biopsies at the preclinical stage are not performed.

In this paper, we performed sequential mRNA-sequencing studies in effector (spleen) and target tissues (kidneys, brain) from lupus and healthy mice, as well as in the whole blood of patients with systemic lupus erythematosus (SLE) (including patients with active or responding LN or neuropsychiatric lupus) and healthy individuals. Comparative cross-tissue and cross-species analyses yielded common, cross-species, nephritis-specific genes that could be prioritised as potential therapeutic targets. Using machine-learning algorithms, we constructed a clinical-transcriptome predictive model that can be tested as a non-invasive ‘liquid biopsy’ marker of kidney disease in patients with SLE, to be used for monitoring of kidney disease in SLE, as well as enrollment in LN prevention and early treatment studies.

## Methods

### Patients and healthy individuals

Patients with SLE (n=261) who met the SLICC 2012 or EULAR/ACR 2019 classification criteria and age-matched and sex-matched healthy individuals (n=106) were recruited from the Departments of Rheumatology and Nephrology at the University Hospitals of Heraklio, ‘Attikon’ University Hospital and the respective Blood Transfusion Units. Active LN was defined by the presence of proteinuria more than 0.5 g/day and active urine sediment. A kidney biopsy was performed in all patients with evidence of active kidney disease. Patients either developed active LN de novo or had had a history of LN and were flaring at the time of sampling. Responding LN was defined by preservation or improvement of kidney function with reduction of proteinuria to less than 50% after 6 months of therapy or less than 0.5–0.7 g/day by 12 months.^20 21^ Following informed consent, whole blood was sampled, and RNA was extracted from all participants.

### Animals

NZB/W-F1 mice were sacrificed at the prepuberty (1 month old), preautoimmunity (3 months old) and nephritic (6 months old with proteinuria more than 200 mg/dL for three consecutive days) stage of SLE. Age-matched C57BL/6 mice were used as controls. Spleen, kidneys and brain were removed for RNA extraction.

### RNA-sequencing

RNA libraries were prepared using the Illumina Truseq kit. Paired-end 37 bp (for mouse) and 67 bp (for human) mRNA-sequencing was performed on the Illumina HiSeq2000 and HiSeq4000, respectively, at the University of Geneva Medical School.^22^ FastQC software assessed quality.^23^ Raw reads were aligned to the mouse (mm10 version) and human (hg38 version) genome using STAR V.2.6 algorithm.^24^ Gene quantification was performed using HTSeq.^25^ Differential expression analysis of mouse and human data was conducted using DESeq2^26^ and edgeR,^27^ respectively. Enrichment and network analyses were performed using gProfiler^28^ and GeneMANIA.^29^ The Expression2Kinases (X2K)^30^ was used to yield transcription factors (TFs), kinases and protein-to-protein interaction (PPI) networks. Prediction of drugs was performed with L1000CDS^2^ search engine.^31^ Statistical significance was set at 5% false discovery rate (Benjamini-Hochberg).

### Machine learning

The human mRNA-sequencing dataset was randomly split into training (70%) and validation (30%) sets. Using the training set and feature selection algorithms, the smallest set of human orthologs that most accurately predicted the outcome of interest was selected. Using these orthologs as predictors, models were fit and compared for their ability to predict human disease. To improve performance, clinical predictors (not included in the definition of active or responding LN) were added to the final model. Accuracy, sensitivity, specificity and area under (AUC) the receiver operating curve (ROC) were determined in the validation set.

Detailed information for all methods can be found in online supplemental material. Scripts used and online supplemental table can be found at https://1drv.ms/u/s!Au_gakpSntTbrGO3-3RQ39ByOId1?e=MLF007.

10.1136/annrheumdis-2021-222069.supp1 Supplementary data

## Results

### Molecular signatures associated with murine LN and transition from preclinical to clinical disease

Patients with SLE are in urgent need for therapeutic interventions targeting molecular pathways enriched within individual tissues to treat their disease effectively and safely. To decipher aberrant molecular pathways enriched uniquely within the kidneys in SLE, we profiled gene expression at the spleen (an effector peripheral lymphoid organ), kidneys and brain (major end-organ tissues) from NZB/W-F1 lupus mice and age-matched C57BL/6 healthy counterparts. Tissues were collected at the clinical (nephritic) stage of the disease when nervous system involvement also occurs. Differentially expressed genes (DEGs) in lupus versus healthy mice tissues were analysed. Using genes differentially expressed within kidneys of the NZB/W-F1 lupus mice but not in other tissues studied, we defined a ‘kidney-specific signature’ comprising 726 DEGs (425 upregulated, 301 downregulated) (online supplemental figure S1A, B, table S1A). Enriched functions within this signature included pathways linked to cell metabolism, innate immune system and neutrophil degranulation (online supplemental figure S1C, table S1B), reiterating the role of neutrophils in lupus kidney injury.^32^ By representing the signature DEGs as a gene network, we found several hub genes with high-degree nodes of the network corresponding to human lupus-susceptibility loci^33–35^ such as *FCGR2B, PTPRC, ITGAM, NCF1* and *RASGRP1* (online supplemental figure S1D, table S1C).

**Figure 1:**
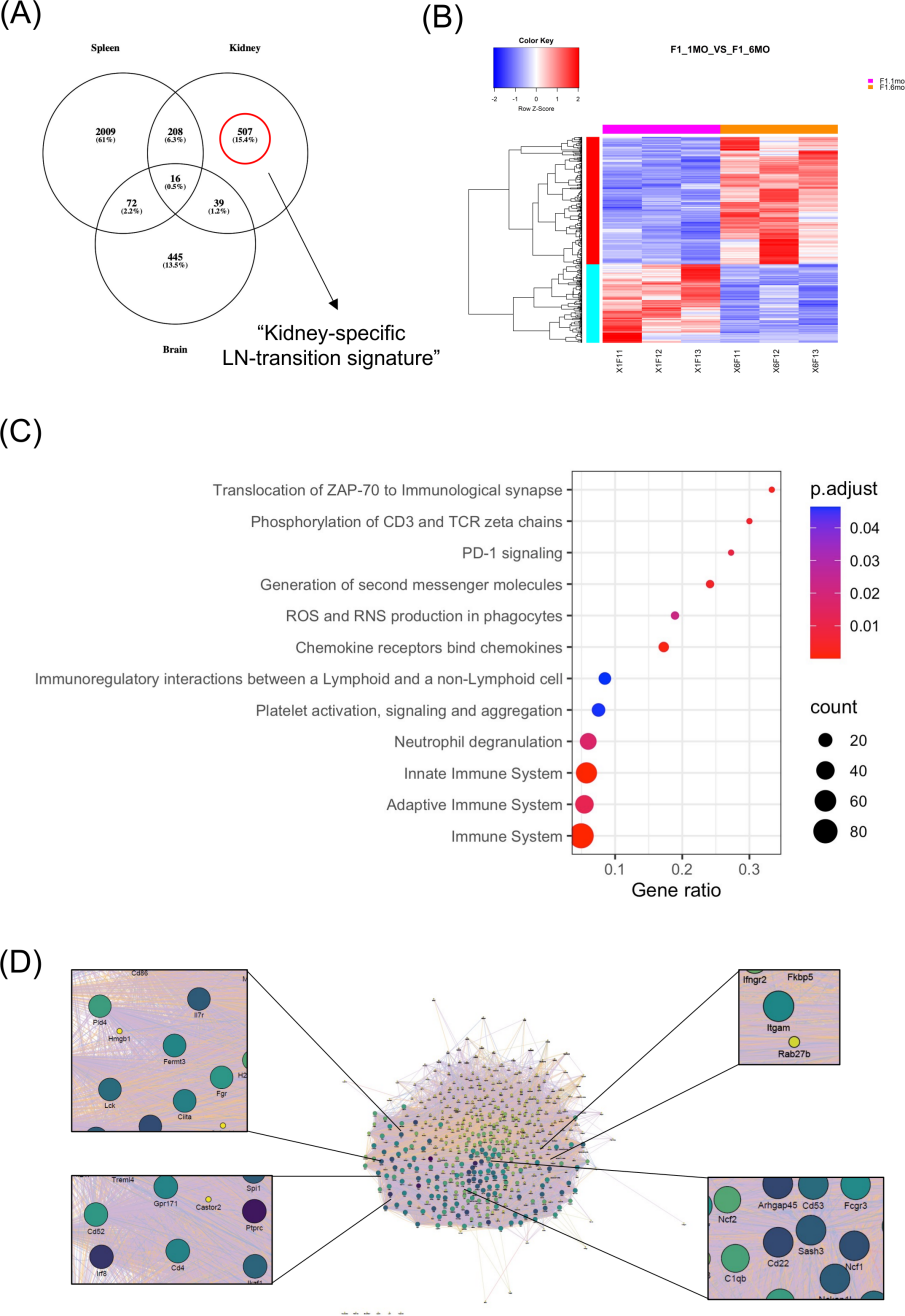
Mouse kidney-specific transcriptome of lupus mice between the clinical (nephritic) and the preclinical (prepuberty) stage of the lupus. (A) Venn diagram demonstrating the comparison between differentially expressed genes (DEGs) within the spleen, the kidneys and the brain from NZB/W-F1 lupus mice at the clinical (nephritic) versus the preclinical (prepuberty) stage of lupus. The kidney-specific gene signature is defined by 507 genes that are differentially expressed only within kidneys but not in other tissues, (B) Heatmap of the 507 kidney-specific DEGs (316 upregulated, 191 downregulated), (C) Dot-plot diagram demonstrating functionally enriched REACTOME pathways of the 507 kidney-specific DEGs, (D) gene network representation of the 507 kidney-specific DEGs. Hub genes that correspond to lupus risk loci are depicted by larger size fonds. ROS, reactive oxygen species; TCR, T cell receptor.

Next, we examined the molecular events underlying transition from the preclinical to clinical stage of lupus kidney disease by comparing DEGs between the tissues from lupus mice probed at the prepuberty versus the nephritic stage. Genes that were differentially expressed uniquely within kidneys of the NZB/W-FI lupus mice but not in other tissues studied defined the ‘kidney-specific LN-transition signature’ comprising 507 DEGs (316 upregulated, 191 downregulated) (figure 1A, B, online supplemental table S2A) that were enriched in innate and adaptive immune system pathways. The former were linked to neutrophil degranulation and reactive oxygen species production in phagocytes, whereas the latter included T cell receptor signalling, signal transduction by G-protein coupled receptors (in particular, chemokine receptors) and costimulation through programmed cell death protein 1 (PD-1) signalling. In addition, pathways involved in platelet activation, signalling and aggregation were identified (figure 1C, online supplemental table S2B). Of note, the lupus-susceptibility risk loci *PTPRC*, *NCF1* and *ITGAM* genes, as well as the *IRF8*,^33–35^ emerged as hub network genes, suggesting a pathogenic role during evolution from preclinical to clinical LN (figure 1D, online supplemental table S2C).

To analyse the sequential molecular events underlying the evolution towards LN, we identified DEGs in tissues from lupus vs healthy mice demonstrating a strain-specific effect in a time-series analysis. DEGs within kidneys demonstrating the lupus-specific pattern were combined with genes within kidneys that were differentially expressed across all stages of the disease. Combined signatures were compared across tissues and genes that were differentially expressed uniquely within kidneys—but not in other tissues—defined the ‘sequential kidney-specific signature’, composed of 1668 genes (online supplemental table S3A). Functional interpretation of the result revealed enrichment in the establishment of sister chromatid cohesion pathway (online supplemental table S3B). Kidney-specific DEGs in lupus versus healthy mice at the preautoimmunity stage, kidney-specific DEGs from lupus mice at the preautoimmunity versus the prepuberty stage and the respective functional enrichment analyses are presented in online supplemental tables S3C–F. DEGs within kidneys demonstrating the strain-specific pattern in the time-series analysis are presented in online supplemental figure S2.

**Figure 2:**
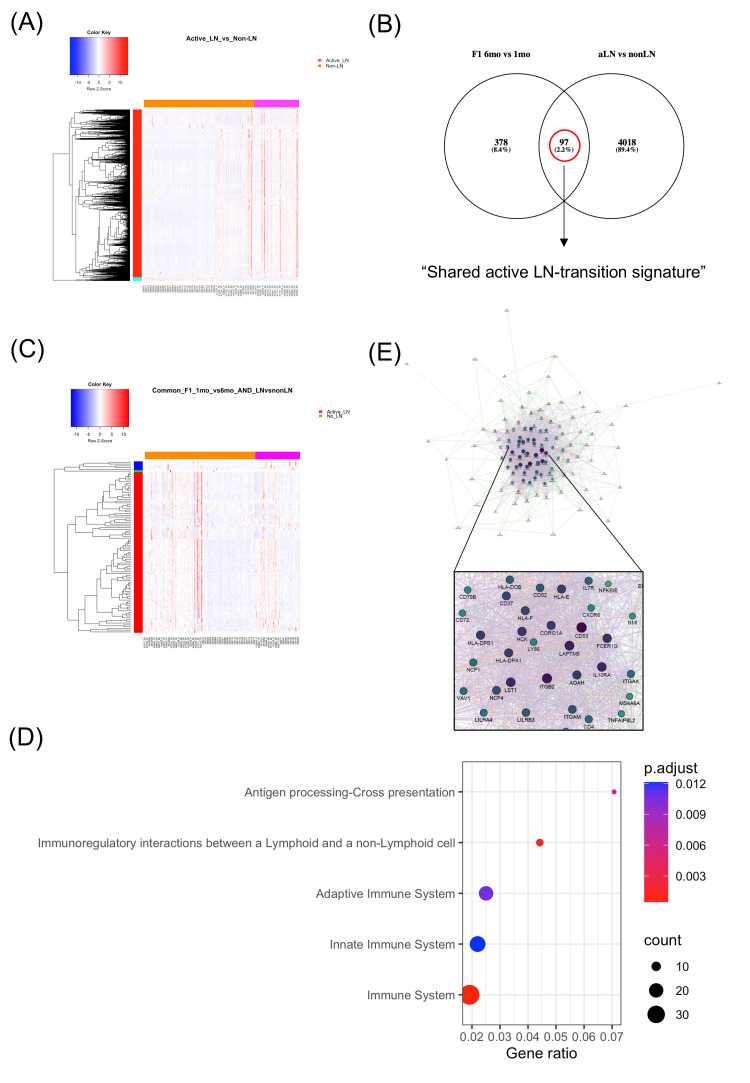
Common genes between the kidney-specific gene expression profile from lupus mice at the symptomatic (nephritic) versus the asymptomatic (prepuberty) stage and the whole-blood gene expression profile from active LN (aLN) patients versus SLE patients without history of kidney involvement (non-LN) define a ‘shared active LN-transition signature’. (A) Heatmap of the 4119 differentially expressed genes (DEGs) in the whole-blood from aLN patients versus non-LN patients, (B) Venn diagram demonstrating the comparison between the orthologous genes of the mouse kidney-specific DEGs from NZB/W-F1 lupus mice at the symptomatic (nephritic) versus the asymptomatic (prepuberty) stage and the whole-blood gene expression profile from aLN versus non-LN SLE patients. The ‘shared active LN-transition signature’ is defined by the union of the Venn diagram, corresponding to 97 common genes, (C) Heatmap of the ‘shared active LN-transition signature’, composed of 97 genes (67 upregulated, 30 downregulated), (D) Dot-plot diagram demonstrating functionally enriched REACTOME pathways of the ‘shared active LN-transition signature’, (E) gene network representation of the ‘shared active LN-transition signature’. Hub genes that correspond to lupus risk loci are depicted by characters of a larger size. LN, lupus nephritis; SLE, systemic lupus erythematosus.

### The human peripheral blood and the murine kidney transcriptome share common kidney-specific signatures and associated hub genes

Kidney biopsy, an invasive procedure linked to increased risk for adverse events, is currently essential to confirm diagnosis and guide therapeutic decisions in LN; however, it is still an imperfect predictor of response to treatment. Previous studies have reported shared molecular signatures within LN kidneys of mice and humans,^36^ as well as between kidney and non-kidney (eg, skin) tissues of patients with LN.^37 38^ Recent evidence suggests that neutrophils from ultraviolet skin reach the kidney and cause inflammation in murine models; it is conceivable that these circulating neutrophils prior to their homing to the kidneys may be captured in the blood.^39^ To this end, we next asked whether the kidney-specific signatures in murine lupus may exist also in patients with LN using blood as an easily accessible, minimally invasive tissue. Specifically, we investigated whether the mouse kidney could serve as non-invasive (not-requiring biopsy in humans) marker of kidney disease in human SLE. To address this, we performed whole-blood mRNA-sequencing in 141 SLE patients and 48 healthy counterparts. Data were combined with our previously analysed cohort,^22^ thus yielding a dataset of 367 individuals (including 261 SLE patients and 106 healthy individuals) (online supplemental table S4A). We found extensive transcriptome perturbations with 10 672 DEGs between active LN patients and healthy individuals (online supplemental figure S3A, table S4B) and 4119 DEGs between active LN and SLE patients without history of kidney disease (non-LN patients) (figure 2A, online supplemental table S4C).

**Figure 3:**
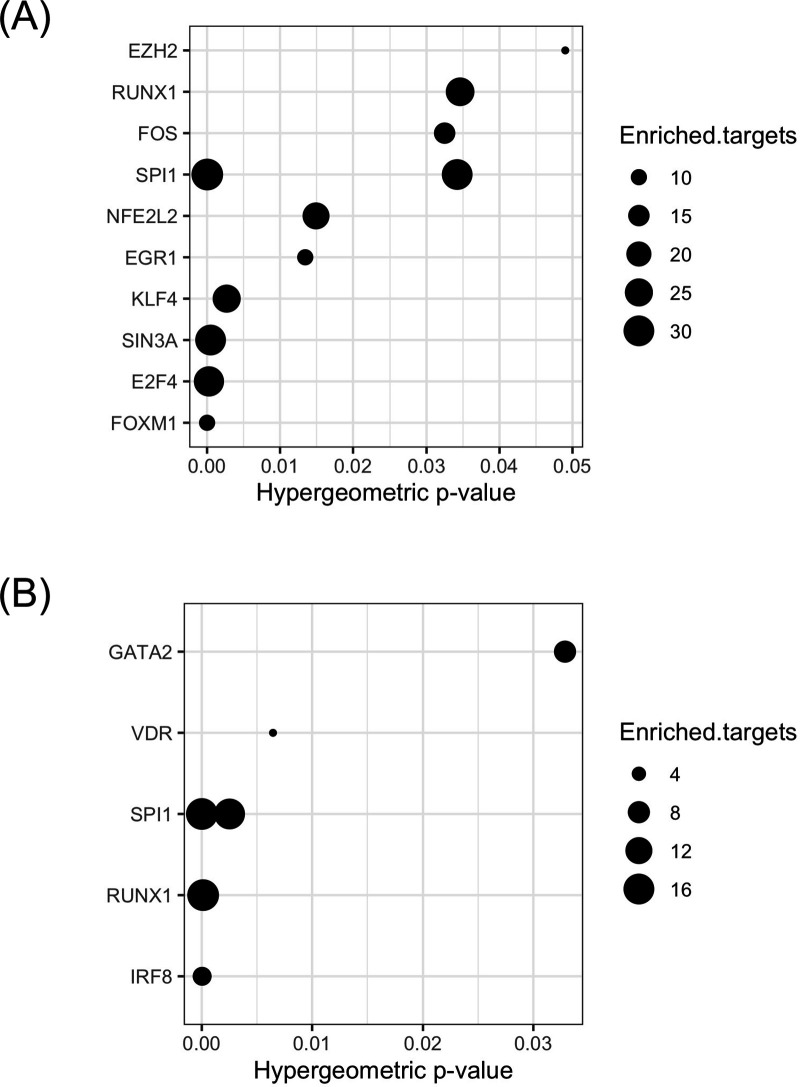
Upstream regulators of the ‘shared active LN signature’ and the ‘shared active LN-transition signature’. (A) Dot-plot diagram demonstrating the transcription factors (TF) that are predicted to reverse the common genes between the kidney-specific gene expression profile from lupus vs healthy mice at the clinical (nephritic) stage and the whole-blood gene expression profile from active LN (aLN) patients vs healthy individuals (HI). The x-axis represents the hypergeometric p value and dots correspond to the number of enriched targets of the TF, (B) Dot-plot diagram demonstrating the TF that are predicted to reverse the common genes between the kidney-specific gene expression profile from lupus mice at the clinical (nephritic) versus the preclinical (prepuberty) stage and the whole-blood gene expression profile from patients with active LN (aLN) versus SLE patients without history of kidney involvement (non-LN). The x-axis represents the hypergeometric p- value and dots correspond to the number of enriched targets of the TF. LN, lupus nephritis; SLE, systemic lupus erythematosus.

Next, we examined whether the human peripheral blood from patients with LN shares common gene expression aberrations with the mouse kidney-specific gene signatures. Using the human orthologous genes of the mouse genome, we examined if the mouse ‘kidney-specific signature’ is present in the blood of patients with active LN as compared with healthy individuals. A total 272 genes (193 upregulated and 79 downregulated) were common between the two datasets (online supplemental figure S3B, C, table S5A), referred to as ‘shared active LN signature’. Neutrophil degranulation was the most significantly enriched pathway in this signature (online supplemental figure S3D, table S5B), whereas gene network analysis revealed that the lupus-susceptibility risk loci *NCF2*, *ITGAM, NCF1, RASGRP1* and *FCGR2A* 33–35 were high-degree hub genes, suggesting their central pathogenic role in LN (online supplemental figure S3E, table S5C).

A similar cross-species analysis was performed to determine whether the mouse ‘kidney-specific LN-transition signature’ intersects with the human blood transcriptome of patients with active LN versus non-LN patients. Ninety-seven common genes (67 upregulated and 30 downregulated) were identified (figure 2B, C, online supplemental table S6A), comprising the ‘shared active LN-transition signature’. Functional enrichment analysis revealed pathways linked to hematopoietic cell lineage, B-cell receptor signalling and immunoregulatory interactions between lymphoid and non-lymphoid cell (figure 2D, online supplemental table S6B). *CD53*, *ITGB2* and *LAPTM5* were the highest-degree hub genes, underscoring their role in evolution of LN. The risk locus *ITGAX* was also identified, further supporting its pathogenic role^33^ and its gene expression deregulation within kidneys during lupus progression (figure 2E, online supplemental table S6C).

To characterise the ‘sequential kidney-specific signature’ in the context of human LN, we compared the human orthologous genes of the mouse signature with the DEGs between active LN patients and healthy individuals and revealed 609 common genes that defined the ‘shared sequential kidney-specific signature’ (online supplemental table S7A). These genes were functionally enriched in pathways linked to selenocysteine synthesis and non-sense mediated decay independent of the exon junction complex (online supplemental table S7B).

In silico analysis of upstream regulators, downstream kinases and drug signatures for the identification of novel therapeutic targets in LN: Kinases TRRAP, AKT2, CDK16 and SCYL1 as putative targets for reversing the LN signature

Genetic association studies have identified TFs to play a major pathogenic role in SLE.^40^ Taking advantage of our study design, we performed TF enrichment analysis^30^ in the cross-species gene signatures and found a total of 11 TFs (including E2F4, FOXM1, SPI1 and SIN3A) and 6 TFs (including SPI1, IRF8, RUNX1 and VDR), which were predicted to regulate the ‘shared active LN signature’ (figure 3A, online supplemental table S8A) and the ‘shared active LN-transition signature’ (figure 3B, online supplemental table S9A), respectively.

To decipher downstream kinases of the shared gene signatures that might serve as druggable targets, the aforementioned lists of enriched TFs were expanded by identifying proteins previously shown to physically interact with them, followed by construction of PPI subnetworks (online supplemental table S8B, table S9B). Based on the overlap between known kinase–substrate phosphorylation interactions and the proteins in the subnetworks, we found kinases that phosphorylate the proteins interacting with the TFs. The kinase TRRAP was predicted to phosphorylate the NCOR2 and HCFC1 (hypergeometric p=0.0004799) that interact with the enriched TFs that regulate the *‘*shared active LN signature’ (online supplemental table S8C); and the AKT2, CDK16 and SCYL1 kinases were predicted to phosphorylate ACTN4 and AES or SMARCA4 or AES (hypergeometric p=0.005443), respectively, that interact with the enriched TFs that regulate the *‘*shared active LN-transition signature’ (online supplemental table S9C), suggesting they could represent putative targets in LN. Complete upstream pathways of the gene signatures connecting the enriched TFs to kinases through known PPIs were also inferred (online supplemental tables S8D and S9D).

Finally, through the L1000 Characteristic Direction Signature Search Engine (L1000CDS^2^), we detected the top 50 drugs or small molecule compounds (online supplemental tables S8E and S9E) and the top 50 compound combinations that may reverse the ‘shared active LN signature’ and the ‘shared active LN-transition signature’, respectively (online supplemental tables S8F and S9F). Among these, the R(+)−6-BROMO-APB was predicted to reverse the former, and the HEMADO, norketamine hydrochloride, trichostatin A and others were predicted to reverse the latter signature, respectively, in the HA1E kidney cell line, suggesting they could be further tested in the therapy of LN.

### Eighteen genes may predict patients with active LN from healthy individuals

**Figure 4:**
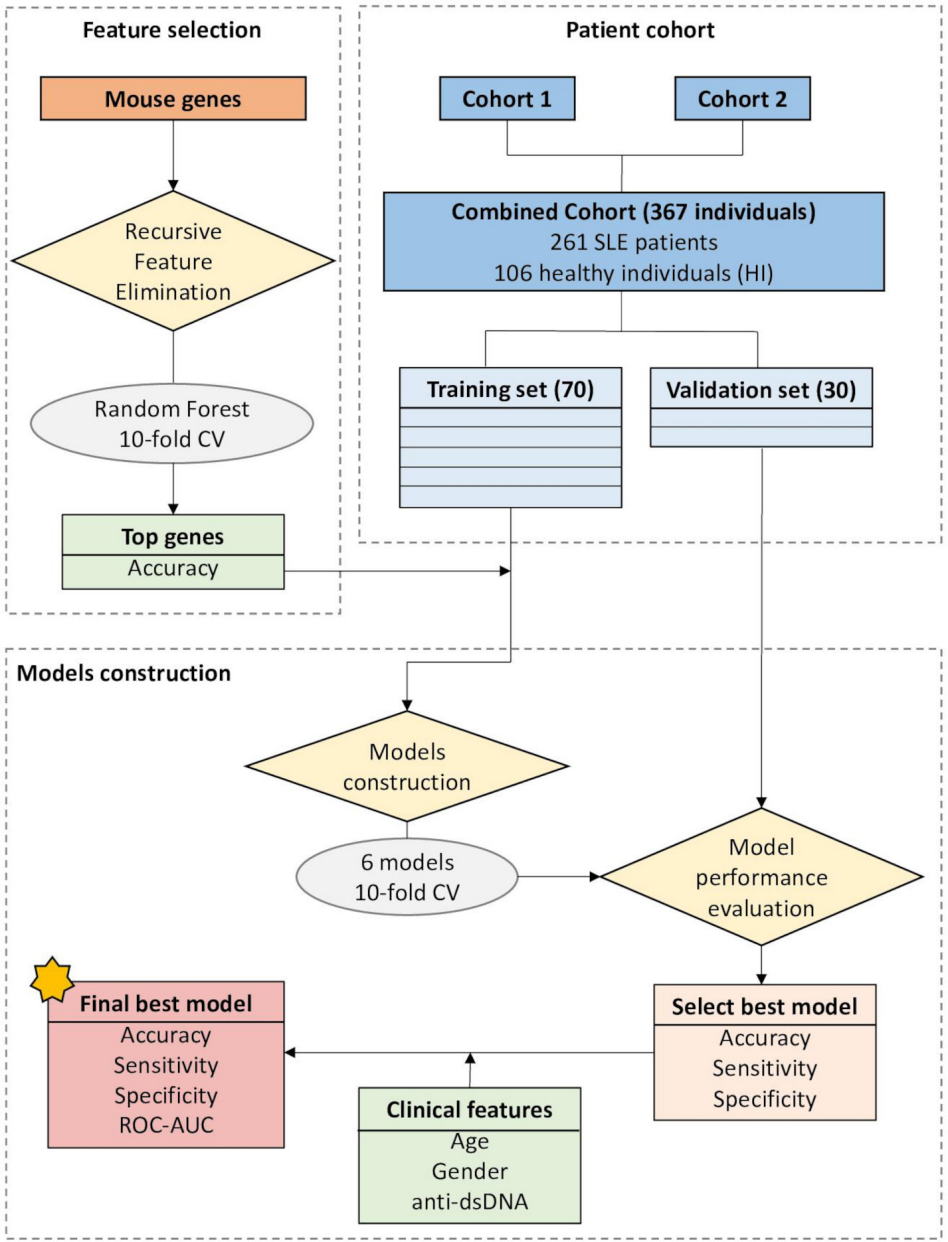
Schematic overview of the machine-learning approach. RNA-sequencing data from the two human cohorts were combined and then split in training to test sets at 70:30 ratio. For each outcome measure, a corresponding gene list derived from mouse data was used. The training set was used to develop a prediction model and the test set was used to validate the results. Using the training set, feature selection was applied to remove noise and keep the smallest set of genes which best predicts each outcome based on accuracy. Then, different prediction models were fit to identify which performs best using the gene signature selected in the previous step. Once the best model was selected based on accuracy, sensitivity and specificity, the addition of age, gender and the presence of anti-dsDNA as predictors were tested if they could improve the model. The final model was validated in the test set. AUC, area under the curve; CV, cross-validation; dsDNA, double-stranded DNA; ROC, receiver operating characteristic curve.

Demographic, clinical and serological data are imperfect in predicting the onset of kidney disease in patients with SLE. Importantly, early identification and prompt treatment have been linked to improved outcomes.^13 14^ We examined whether the human orthologs of the mouse kidney-specific gene signatures and the human whole-blood gene signatures may predict those patients with SLE who will develop LN. For this, the complete mRNA-sequencing dataset was randomly split into training (70%) and validation (30%) sets, and machine-learning algorithms were applied (figure 4).

**Figure 5:**
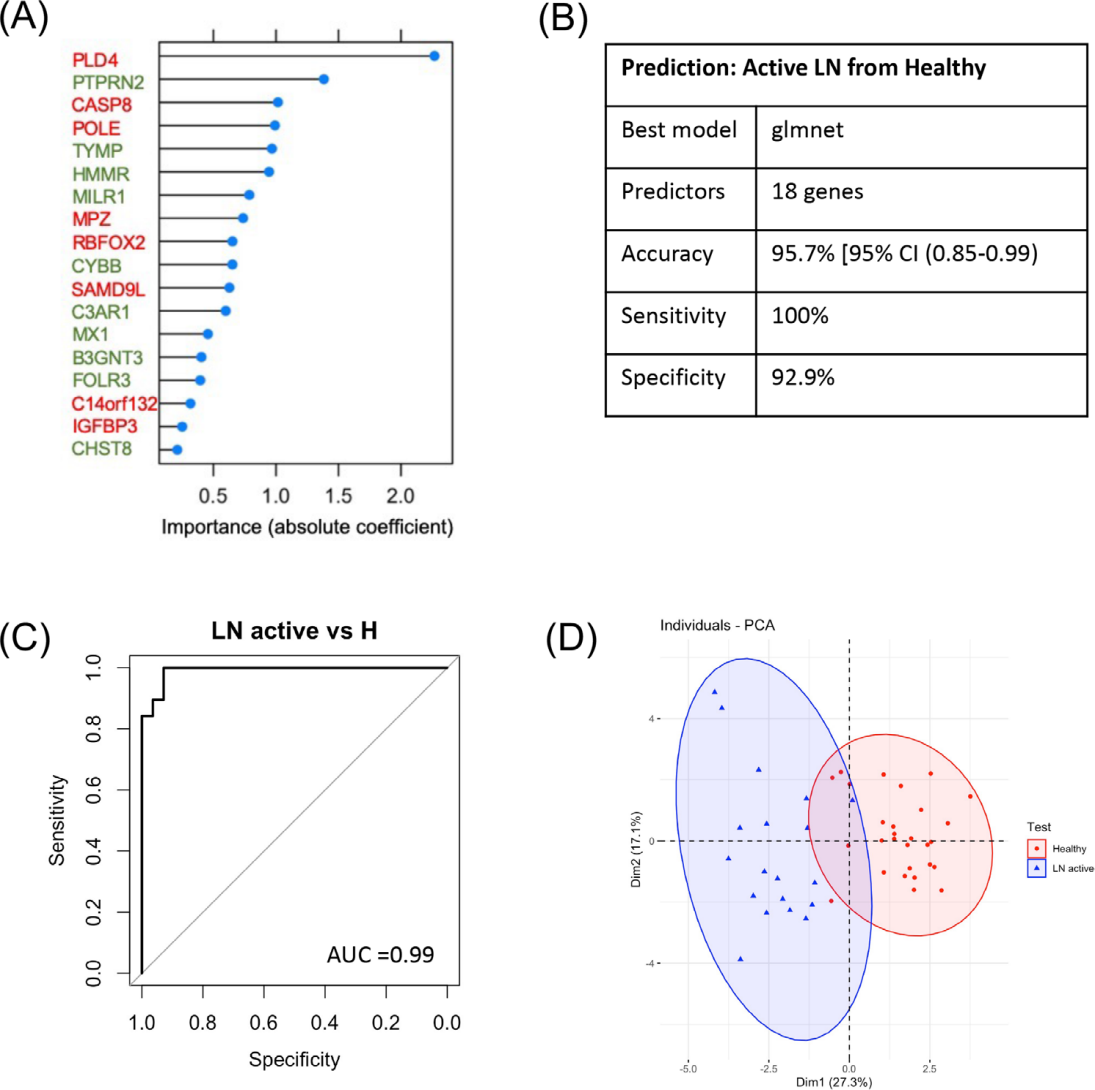
Machine-learning modelling of the human whole-blood RNA-sequencing data, using mouse kidney-specific genes as predictors, distinguishes patients with active lupus nephritis (active LN) from healthy individuals (H) in a non-invasive manner and defines a LN prognostic gene signature. (A) The 18 predictors of the glmnet model distinguishing patients with active LN from healthy individuals based on their importance, as evidenced by their absolute coefficient. Gene predictors in green fonts indicate that the higher their expression the higher the probability of being a patient with active LN compared with being a healthy individual; while gene predictors in red fonts indicate that the lower their expression the higher the probability of being a patient with active LN, (B) Characteristics of the prediction model of patients with active LN from healthy individuals, (C) Receiver operating characteristic curve (ROC) analysis of the glmnet model in the validation set reveals an area under the curve (AUC) of 0.99, (D) principal component analysis (PCA) using the 18 genes.

To distinguish patients with active LN from healthy individuals, we used the human orthologs of the mouse kidney-specific DEGs from lupus versus healthy mice at the nephritic stage (corresponding to the ‘kidney-specific signature’, composed of 726 DEGs). To remove noise and keep the smallest set of human orthologs of the mouse genes which best predicts outcome, we performed feature selection using recursive feature elimination with a random forest (machine-learning) model under a 10-fold cross-validation. Based on model accuracy, a set of 50 human orthologs were selected. Next, prediction models were fit to identify which performs best with the selected genes. The glmnet model using 18 genes—including *PLD4*, *PTPRN2*, *CASP8* and *POLE* (figure 5A, online supplemental table S10)—(32 genes had a coefficient=0 and were considered redundant in the model) best distinguished patients with active LN from healthy individuals with a 10-fold cross-validation calculated accuracy of 95.7% (95% CI (0.85% to 0.99%)], 100% sensitivity and 92.9% specificity (0.99 AUC of the ROC curve analysis) in the validation set (figure 5B, C), demonstrating an excellent model efficiency to discriminate true positive (active LN patients) from false positive (healthy individuals) cases. Inclusion of clinical factors (not included in the definition of active or responding LN), such as age, gender and the presence of anti-dsDNA, did not improve further the performance of the model. Using the validation set, principal component analysis (PCA) demonstrated that the 18 selected genes could accurately discriminate patients with active LN from healthy individuals (figure 5D). The relationship between the expression of each gene and the probability of predicting active LN is demonstrated in online supplemental figure S4. These data define a LN prognostic gene signature and demonstrate the feasibility of developing and validating an algorithm to predict patients with active LN from healthy individuals non-invasively, through machine-learning analysis of a large whole blood RNA-sequencing dataset of SLE patients using human orthologs of mouse kidney-specific genes as predictors of kidney involvement.

### Machine-learning model distinguishes LN from non-LN SLE patients

**Figure 6:**
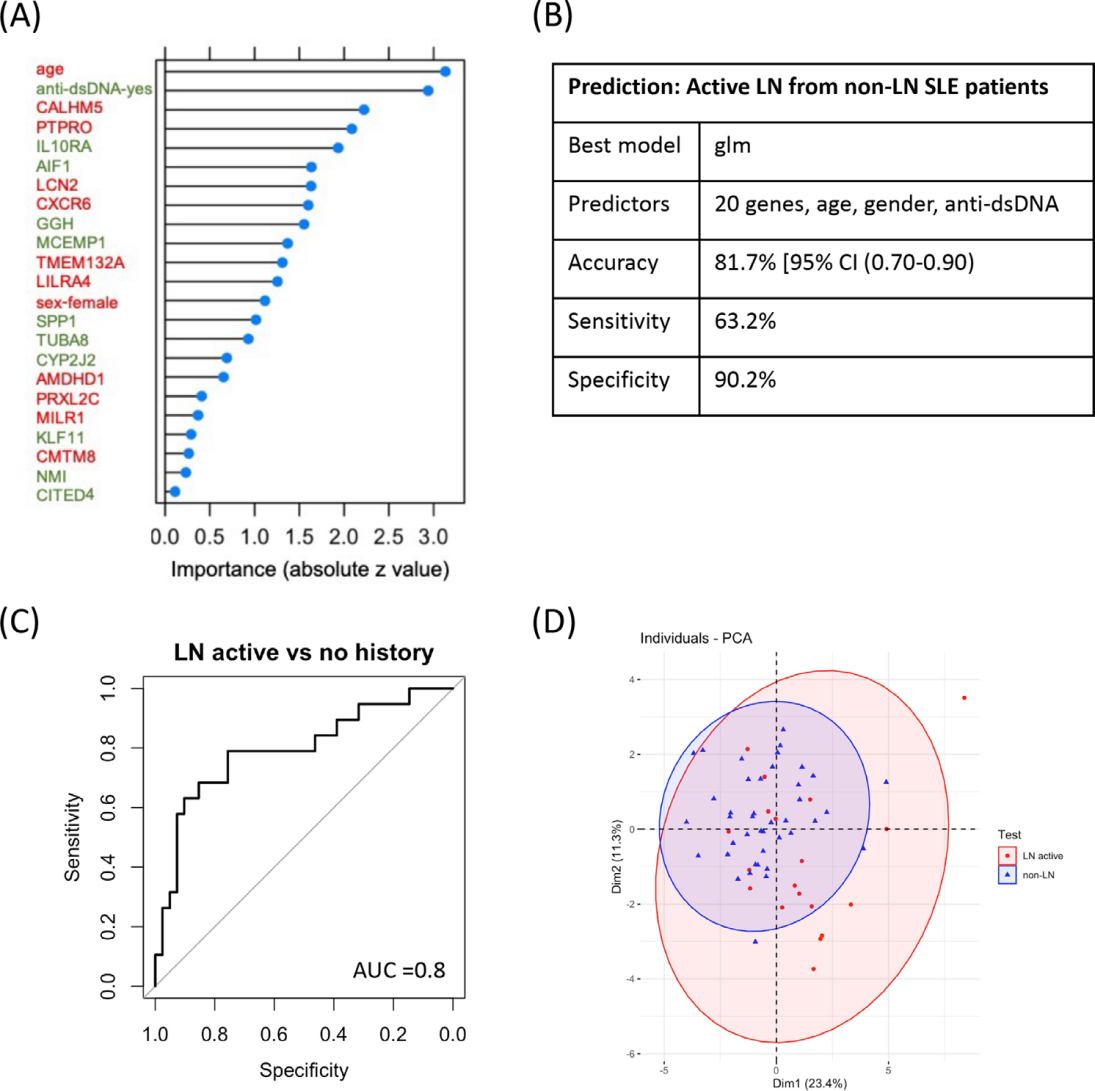
Machine-learning modelling of the human whole-blood RNA-sequencing data using mouse kidney-specific LN-transition genes as predictors distinguishes patients with active lupus nephritis (active LN) from SLE patients without history of kidney disease, non-invasively. (A) The 23 predictors of the glm model distinguishing patients with active LN (active LN) from SLE patients without kidney disease (non-LN) based on their importance, as evidenced by absolute z value. Gene predictors in green fonts indicate that the higher their expression the higher the probability of being a patient with active LN compared with being non-LN patient, while gene predictors in red fonts indicate that the lower their expression the higher the probability of being a patient with active LN. The presence of anti-dsDNA (indicated in green fonts) is associated with a higher the probability of being a patient with active LN and the older age and female gender (indicated in red fonts) are associated with a lower probability of being a patient with active LN, (B) Characteristics of the prediction model of active LN patients from non-LN patients, (C) Receiver operating characteristic curve analysis of the glm model in the validation set reveals an area under the curve (AUC) of 0.8, (D) Principal component analysis (PCA) using the 20 gene-predictors. LN, lupus nephritis; SLE, systemic lupus erythematosus;

Next, we examined whether the above approach could also discriminate active LN patients from SLE patients without kidney disease (non-LN patients) in a non-invasive manner. We sought that the kidney-specific gene expression profile of lupus mice at the clinical (nephritic) versus the preclinical (prepuberty) stage of the disease (corresponding to the ‘kidney-specific LN-transition signature’, composed of 507 DEGs) could reflect the whole-blood gene expression profile of SLE patients with active LN versus SLE patients without history of LN (non-LN patients). Thus, we used the human orthologs of the mouse ‘kidney-specific LN-transition signature’ as predictors, and applied feature selection under a 10-fold cross-validation. Based on accuracy, 20 genes best predicted the outcome. Models were fit to identify which performs best with the selected genes. Model performance was further improved by the addition of age, sex and presence of anti-dsDNA, as predictors of outcome. As expected, due to the higher likelihood of patients with proliferative LN to have anti-DNA antibodies, the presence of anti-dsDNA was the most important predictor of kidney disease, followed by the expression of *PTPRO* gene (the lower its expression, the higher the probability of predicting active LN) and *IL10RA* gene (the higher its expression, the higher the probability of predicting active LN). Male sex and younger age of SLE patients were associated with higher probability of active LN. In the validation dataset, the glm model displayed accuracy 81.7% (95% CI (0.70% to 0.90%)), sensitivity 63.2% and specificity 90.2% (AUC 0.80) in distinguishing patients with active LN from SLE patients without history of LN (figure 6A–C, online supplemental table S11, figure S5), demonstrating that the model correctly identified SLE patients without LN (true negative cases). Using the validation set, PCA demonstrated how gene predictors could accurately discriminate patients with active LN from non-LN SLE patients (figure 6D). Together, these data demonstrate the feasibility to distinguish patients with active LN from SLE patients without kidney involvement. These gene predictors could be of prognostic value in the clinical setting following further validation studies in independent cohorts.

## Discussion

Patients with LN are in need for an early diagnosis and therapeutic targeting of aberrant molecular pathways enriched within the affected kidneys. Here, we performed sequential mRNA-sequencing in three tissues of lupus and healthy mice, and in the whole-blood of SLE and healthy individuals. Through cross-tissue analysis, we defined a murine kidney-specific molecular signature and a molecular signature that underlines progression from the predisease stage to overt clinical disease. We also demonstrated that the murine kidney transcriptome mirrors—in part—the human whole blood transcriptome of LN patients and found upstream and downstream transcriptional regulators that may be prioritised as potential therapeutic targets. Finally, we developed a blood gene-based predictive model for human LN that can be tested as an alternative, non-invasive ‘liquid biopsy’ marker of kidney disease in patients with SLE. Pending further confirmation, this marker could identify patients in need of monitoring for development of LN, as well as enrolment in LN prevention and early treatment studies.

To improve therapeutic interventions and optimise the use of animal models, gene expression profiling across three samples and species is important in defining how mouse biology can be extrapolated to humans.^41^ To this end, the sequential cross-organ (murine spleen, kidney and brain) and cross-species (murine and human) comparative transcriptomics analysis in this paper is novel, defining unique-to-kidney molecular aberrancies in SLE that can be extrapolated to the transition from the preclinical to clinical stage of human LN. Our human transcriptomic analysis involved a large number of well-characterised patients and healthy controls which makes it the larger, single-centre, RNA-seq analysis ever performed in SLE. In addition to providing potential biomarkers for prediction and non-invasive diagnosis and monitoring, our data also reflect biological pathways involved both in the development and clinical transition of LN in a systematic and unbiased manner, without preconceived notions.

In view of the heterogeneity of lupus, we used next-generation sequencing as an unbiased and not requiring a priori hypothesis approach to uncover novel molecular pathways implicated in major end-organ injury in SLE. Initially we performed mRNA-sequencing of a peripheral lymphoid organ (the spleen, that may be used as a surrogate of peripheral blood) and two end-organ tissues (kidneys and brain) from the NZB/W-F1 lupus model at the prepuberty, preautoimmunity and nephritic stage of SLE and identified the molecular profile which is expressed uniquely within kidneys of this model—but not in other tissues studied—and the molecular profile that characterises unique-to-kidney molecular events underlying LN transition from the preclinical to clinical stage of kidney disease. In this process, we identified pathways enriched within each signature and found that hub genes correspond to lupus susceptibility risk loci (such as the *PTPRC, ITGAM, NCF1* and *IRF8* genes), reinforcing their pathogenic role in LN and the progression from preclinical to clinical kidney disease. Validating our results, the *VEGF, TLR2* and *SOCS3* genes were also differentially expressed in the kidneys from NZB/W-F1 mice 9 months old vs 6 months old as well as the kidneys from patients with LN.^36^ In agreement with Arazi *et al*,^42^ genes such as the *ITGAM* and *FCGR2B* were also differentially expressed in the ‘kidney-specific gene signature’. The FPR2, *IL18R1*, *ITGAM* and *NCF4* genes were also differentially expressed in the myeloid lineage from paediatric patients with LN,^43^ genes such as the *MDP1*, *PTGR1* and *MX2* were also differentially expressed within the kidneys from LN patients, as assessed by microarrays^44^ and genes such as the *TMEM167A*, *TNFAIP8* and *VCAM1* were also differentially expressed in kidney tubular cells from LN patients.^38^

Blood transcriptome analysis identified similarities as well as differences from the molecular signatures detected within kidneys in patients with LN, underscoring that limitations exist in the use of blood for uncovering kidney disease processes.^42^ However, gene expression studies have shown shared inflammatory responses within kidneys between mice and humans with LN,^36^ but also shared gene signatures between kidney tubular cells and keratinocytes of LN patients.^37 38^ Our data suggest that the mouse kidney transcriptome and the human whole-blood transcriptome share a common gene expression profile that corresponds to common biological processes and pathways. Lupus medications were held for 12 hours prior to sampling thus, a potential downstream effect cannot be excluded. However, validating our results, in the ‘shared active LN signature’, genes such as the *CEACAM1*, *TYMP, NCOA7* and *AIM2* were also differentially expressed in interferon stimulating genes identified through single-cell RNA-sequencing within the kidneys from LN patients^42^ and *SERPINA1*, *IL1RN* and *ABCB1* genes were also differentially expressed in kidney tubular cells from LN patients.^38^ We also identified hub genes of the common cross-species kidney-specific gene network corresponding to lupus-susceptibility risk loci, uncovering their cross-species pathogenic role in LN, and identified that the pathway interactions between lymphoid and non-lymphoid cell characterises the transition from preclinical to clinical LN across species. Although we do not validate the LN blood transcriptome with the kidney transcriptome in humans, part of the mouse kidney transcriptome mirrors the human whole-blood transcriptome in patients with LN, suggesting that common genes can be prioritised as potential therapeutic targets for LN, or tested as an alternative, non-invasive ‘liquid biopsy’ marker of kidney disease in patients with SLE.

To decipher cross-species specific targets in LN, we used systems biology approaches and combined our experimental data with simulation-based analyses. We report upstream and downstream regulators of the cross-species kidney-specific gene signatures as specific targets in LN and describe novel cross-species drug signatures for kidney disease in lupus, suggesting non-immune-based approaches to be tested in LN therapeutics, as ‘add on’ therapy to conventional immune therapy. We must underscore that due to limitations in the analysis, identified TFs are not restricted to immune cells therefore therapies targeting them could have off-target effects with potential toxicity.

Although current therapeutic decisions in LN are guided by its histological classification,^20 21 45^ kidney histology is an imperfect predictor of kidney outcome,^1^ highlighting the need for improved biomarkers.^44^ The urokinase-type plasminogen activator receptor and the decrease in urinary epidermal growth factor to creatine ratio have been identified as independent predictors of progression to chronic kidney disease in patients with glomerular diseases^46 47^; however, a biomarker for preclinical LN has not been identified. Since preclinical LN is an early stage in the natural history of the disease and improvements in the prognosis of LN have been attributed to early diagnosis and prompt therapy,^10–14^ we used machine-learning approaches to identify non-invasive predictors of kidney involvement in SLE patients. Specifically, we used the ‘kidney-specific gene signature’ as a tool to build a machine-learning algorithm to distinguish patients with active LN from healthy individuals and demonstrated that this approach can be used successfully as a non-invasive prediction method. Then, using the murine lupus kidney-specific transcriptome, we built and validated a machine-learning algorithm that predicts patients with active LN from SLE patients without LN, to be used in the monitoring for kidney disease in such patients and enrolment in LN prevention and early treatment studies. Although validation in an independent dataset was not used, cross-validation was performed during modelling, thus reinforcing our results. These gene predictors could be of prognostic value in the clinical setting, following further validation studies in independent cohorts. Although machine-learning distinguishes patients with LN from non-LN patients accurately, yet at this point this method is not better than clinical diagnosis of LN. Moreover, sequential clinical and transcriptomic data are necessary for the prediction of patients that will flare. The prediction of patients that truly have responding LN would have also been useful; however, a kidney-specific signature corresponding to responding kidney disease (not preclinical) is not available in murine, making this algorithm not applicable for this purpose. Further validation in independent human datasets or longitudinal studies are needed to further explore these findings in human LN.

In conclusion, common cross-species, nephritis-specific genes could be used as potential therapeutic targets for LN or tested as a surrogate, non-invasive ‘liquid biopsy’ marker of kidney disease in patients with SLE. These kidney-specific genes can be used to design prevention and early intervention trials, following their validation in longitudinal studies.

## Data Availability

Data are available on reasonable request.
