# Supplementary material for: Cross-species transcriptome analysis for early detection and specific therapeutic targeting of human lupus nephritis
Source: Ann Rheum Dis. 2022 Jul 29;81(10):1409–19. doi: 10.1136/annrheumdis-2021-222069 (PMC9484391; doi:10.1136/annrheumdis-2021-222069)
Supplement: Supplementary data [file annrheumdis-2021-222069supp001.pdf]

ONLINE SUPPLEMENTARY MATERIAL

METHODS

Patients and healthy individuals

Patients with SLE were enrolled from the Departments of Rheumatology or Nephrology of the University Hospital of Heraklion and "Attikon" University Hospital in Athens. A protocol for patient clinical assessment, blood sampling and storage was followed. *Inclusion criteria* included: (a) fulfilment of the revised 1997 ACR classification criteria for SLE(1) and (b) present or past history of moderate/severe activity from SLE requiring the use of immunosuppressive/cytotoxic treatment (azathioprine, mycophenolate, cyclophosphamide). The study was designed to include patients with: (a) *inactive SLE* (remission), defined as clinical SLEDAI-2K=0 on prednisone 0–5 mg/day, allowing for stable dose of immunosuppressants and/or and antimalarials(2) (b) *active SLE*, defined as clinical SLEDAI>0 and PGA (Physician Global Assessment) >0.5. A subset of the latter group met the definition of Low Lupus Disease Activity State (LLDAS)(3). *Exclusion criteria* included: (a) receive of morning glucocorticoid and/or immunosuppressive treatment (b) recent (within the last month) treatment with pulse intravenous methylprednisolone or cyclophosphamide (c) pregnancy (d) active infection or malignancy (e) concomitant autoinflammatory or rheumatic disease. Active LN was defined by the presence of proteinuria more than 0.5 g/day and active urine sediment [denoted by the presence of dysmorphic RBCs (such as acanthocytes) and/or RBC casts and/or WBCs/WBC casts in urine sediment in the absence of infection]. A kidney biopsy was performed in all patients with evidence of active kidney disease. These patients either developed active LN *de novo* or had had a history of LN and were flaring at the time of sampling. Responding LN was defined by preservation or improvement of kidney function with reduction of proteinuria to less than 50% after 6 months of therapy or less than 0.5-0.7 g/day by 12 months. Age- and sex-matched healthy individuals were recruited from the respective participating Blood Transfusion Units (**table S4A**). Informed consent was obtained from participants. Procedures were approved by the local institutional review boards. Assays were performed in whole blood (PaxGene RNA tubes for mRNA extraction, EDTA tubes for DNA extraction). Patients withdrew lupus medications for 12 hours prior to sampling(4–6).

Animals

New Zealand Black (NZB) female mice were crossed with New Zealand White (NZW) male mice at the Animal Facility of the Biomedical Research Foundation of the Academy of Athens (BRFAA). Female hybrids NZB/W-F1(7)(8)(9) (n=3 at each time-point) -the oldest spontaneous murine lupus model that fully recapitulates the human lupus nephritis- were sacrificed with perfusion with PBS at the pre-puberty stage (1 month old), pre-autoimmunity (3 months old) and nephritic stage (6 months old where proteinuria was more than 200 mg/dl for 3 consecutive days) of the disease. Spleen, kidneys and brain were extracted. Age-matched female C57BL/6 mice (n=3) were used as controls. The study and all procedures were approved by the local institutional review board.

RNA sequencing

Mouse samples: Total RNA was extracted using TRIzol reagent (Invitrogen) and libraries were prepared using the Illumina Truseq RNA Sample Prep kit v2. Paired-end 37-bp mRNA sequencing was performed on Illumina HiSeq2000 at the Department of Genetic Medicine and Development of the Medical School of the University of Geneva.

Human samples: Total RNA was extracted using Paxgene Blood miRNA kit (PreAnalytiX) and libraries were prepared using the Illumina stranded Truseq mRNA protocol. Paired-end 67bp mRNA sequencing was performed on Illumina HiSeq4000 at the Department of Genetic Medicine and

Development of the Medical School of the University of Geneva. Library preparation and sequencing for the published human dataset are reported in reference(5).

Data processing: Quality of sequencing was assessed using FastQC software(10). Raw reads in fastq format were collected and aligned to the mouse genome (mm10 version) and human genome (hg38 version) using STAR 2.6 algorithm(11). Gene quantification was performed using HTSeq using the -s reverse option for stranded data(12). Gencode annotation file version 29 was used for the annotation of human data and version M19 for mouse data. Published data in fastq format was processed as reported above.

Differential gene expression and computational analysis of RNA-sequencing data

Differential expression analysis of mouse data was conducted using DESeq2(13) and differential expression analysis of human data using edgeR packages in R(14). Clustering of genes was performed using Euclidean distance. Unsupervised analysis was used to assess whether similarities and distinctions between samples were present. To identify sequentially differentially expressed signatures, all differentially expressed genes (DEGs) with a strain-specific pattern across time were combined. To do this, a time-series analysis -where multiple terms were tested at once- was performed. Specifically, a "full model" including the time-factor was developed to examine for the presence of any difference over the 3 stages of the disease, and a likelihood ratio test assessed for the presence of any difference over the 3 stages of the disease in a "reduced model" where the time-factor was removed. Statistically significant DEGs at one or more stages of the disease after the pre-puberty stage, determined by the difference in deviance between the "full" and "reduced" model formula, demonstrated a strain-specific effect. Enrichment analysis of DEGs, visualization and network analysis were carried out using gProfiler(15) and GeneMANIA(16). The Expression2Kinases (X2K)(17) was used to yield upstream and downstream regulators ($ARCHS^4$) and PPI networks. For all statistical comparisons, the cut-off for significance was set at 5% false discovery rate (FDR; Benjamini-Hochberg). Heatmaps were created using heatmap3 package(18) in R. Prediction of drugs which reverse observed gene signatures was carried out by $L1000CDS^2$ search engine(19).

Machine learning

RNA-sequencing data from both human cohorts were combined and then split in training:test sets (70:30 ratio). Pre-processing of raw counts (vst transformation) was performed separately for the training and the test set using DESeq2(13). The transformed counts of the test set were calculated based on the parameters of the training set. The training set was used to develop a prediction model and the test set was used to validate the results using caret(20) package in R. For each outcome measure, a corresponding gene list derived from mouse data was used. Using the training set, recursive feature elimination (RFE) using 5 repeats of 10-fold cross-validation (CV) was used as a feature selection step (random forest model) to remove noise and keep the smallest set of genes which best predicts each outcome based on accuracy. Then, collinearity between the features including age, gender and dsDNA was checked using *findCorrelation* function from caret package. Highly correlated features (>0.75 correlation coefficient) were excluded. Next, six different prediction models (rf, glm, glmnet, svmRadial, svmPoly, nnet from caret package) were fit to identify which performs best using the gene signature selected in the previous step using 5 repeats of 10-fold CV. The best model was selected based on accuracy, sensitivity, and specificity. Once the best model was selected it was tested whether the addition of age, gender and anti-dsDNA as predictors improve the model (parameters included in the definition of active or responding LN were not further tested as predictors). The final model was validated in the test set. Accuracy, sensitivity, and specificity of the test set are reported. ROC curves were generated using the pROC package(21) in R and PCA plots using FactoMineR(22). The importance of each feature in the final predictive models was calculated using *varImp* function

from caret package. The association (positive or negative) of each feature with the predicted outcome was evaluated using Partial Dependence Plots (PD plots) using *pdp* package(23) in R.

Data Sharing Statement

Murine RNA-seq data have been deposited to GEO (accession number GSE186367). Human RNA-seq data have been deposited to EGA database under Study EGAS; dataset EGAD (accession number EGAS00001005701).

SUPPLEMENTARY TABLE LEGENDS

Table S1A. Kidney-specific differentially expressed genes (DEGs) between lupus vs healthy mice at the clinical (nephritic) stage of the disease

Table S1B. Functional enrichment analysis of differentially expressed genes (DEGs) between lupus vs healthy mice at the clinical (nephritic) stage of the disease

Table S1C. Gene network representation of differentially expressed genes (DEGs) between lupus vs healthy mice at the clinical (nephritic) stage of the disease

Table S2A. Kidney-specific differentially expressed genes (DEGs) between the clinical (nephritic) vs the pre-clinical (pre-puberty) stage of the disease from lupus mice

Table S2B. Functional enrichment analysis of differentially expressed genes (DEGs) between the clinical (nephritic) vs the pre- clinical (pre-puberty) stage of the disease from lupus mice

Table S2C. Gene network representation of differentially expressed genes (DEGs) between the clinical (nephritic) vs the pre-clinical (pre-puberty) stage of the disease from lupus mice

Table S3A. Kidney-specific differentially expressed genes (DEGs) defining the "*sequential kidney-specific signature*"

Table S3B. Functional enrichment analysis of kidney-specific differentially expressed genes (DEGs) defining the "*sequential kidney-specific signature*"

Table S3C. Kidney-specific differentially expressed genes (DEGs) in lupus *versus* healthy mice at the pre-autoimmunity stage

Table S3D. Kidney-specific differentially expressed genes (DEGs) from lupus mice probed at the pre-autoimmunity *versus* the pre-puberty stage

Table S3E. Functional enrichment analysis of kidney-specific differentially expressed genes (DEGs) in lupus *versus* healthy mice at the pre-autoimmunity stage

Table S3F. Functional enrichment analysis of kidney-specific differentially expressed genes (DEGs) from lupus mice probed at the pre-autoimmunity *versus* the pre-puberty stage

Table S4A. Demographics and clinical characteristics of patients with SLE and healthy individuals

Table S4B. Differentially expressed genes (DEGs) within the peripheral whole-blood of patients with active lupus nephritis vs healthy individuals

Table S4C. Differentially expressed genes (DEGs) within the peripheral whole-blood of patients with active lupus nephritis vs SLE patients without history of kidney involvement (non-LN patients)

Table S5A. Common genes between the orthologous genes of the mouse kidney-specific differentially expressed genes (DEGs) from lupus vs healthy mice and the whole-blood DEGs from patients with active lupus nephritis (aLN) vs healthy individuals (HI) ("shared active LN signature")

Frangou E, *et al. Ann Rheum Dis* 2022; 81:1409–1419. doi: 10.1136/annrheumdis-2021-222069

Table S5B. Functional enrichment analysis of the “shared active LN signature”

Table S5C. Gene network representation of the “shared active LN signature”

Table S6A. Common genes between the orthologous genes of the mouse kidney-specific differentially expressed genes (DEGs) between the clinical (nephritic) vs the pre-clinical (pre-puberty) stage of the disease from lupus mice and the whole-blood DEGs from patients with active lupus nephritis (aLN) vs SLE patients without history of kidney involvement (non-LN) (“shared active LN-transition signature”)

Table S6B. Functional enrichment analysis of the “shared active LN-transition signature”

Table S6C. Gene network representation of the “shared active LN-transition signature”

Table S7A. Differentially expressed genes (DEGs) defining the “*shared sequential kidney-specific signature*”

Table S7B. Functional enrichment analysis of the differentially expressed genes (DEGs) defining the “*shared sequential kidney-specific signature*”

Table S8A. Transcription factors (TFs) that are predicted to reverse the “shared active LN signature”

Table S8B. Protein-to-protein interaction (PPI) subnetwork of the enriched transcription factors (TFs) of the “shared active LN signature”

Table S8C. Kinases that are predicted to phosphorylate the PPI subnetwork of the enriched transcription factors (TFs) of the “shared active LN signature”

Table S8D. Upstream pathway of the “shared active LN signature” connecting the enriched TFs to kinases through PPI

Tables S8E. Top-50 drugs or small molecules that are predicted to reverse the “shared active LN signature”

Table S8F. Top-50 compound combinations that are predicted to reverse the “shared active LN signature”

Table S9A. Transcription factors (TFs) that are predicted to reverse the “shared active LN-transition signature”

Table S9B. Protein-to-protein interaction (PPI) subnetwork of the enriched transcription factors (TFs) of the “shared active LN-transition signature”

Table S9C. Kinases that are predicted to phosphorylate the PPI subnetwork of the enriched transcription factors (TFs) of the “shared active LN-transition signature”

Table S9D. Upstream pathway of the “shared active LN-transition signature” connecting the enriched TFs to kinases through PPI

Table S9E. Top-50 drugs or small molecules that are predicted to reverse the “shared active LN-transition signature”

Table S9F. Top-50 compound combinations that are predicted to reverse the “shared active LN-transition signature”

Table S10. Gene predictors that distinguish patients with active LN from healthy individuals

Table S11. Predictors that distinguish patients with active LN from SLE patients without history of kidney involvement.

Frangou E, *et al. Ann Rheum Dis* 2022; 81:1409–1419. doi: 10.1136/annrheumdis-2021-222069

SUPPLEMENTARY TABLES AND SCRIPTS USED CAN BE FOUND AT

https://1drv.ms/u/s!Au_gakpSntTbrGO3-3RQ39ByOId1?e=MLF007

SUPPLEMENTARY FIGURES AND LEGENDS

Fig. S1.

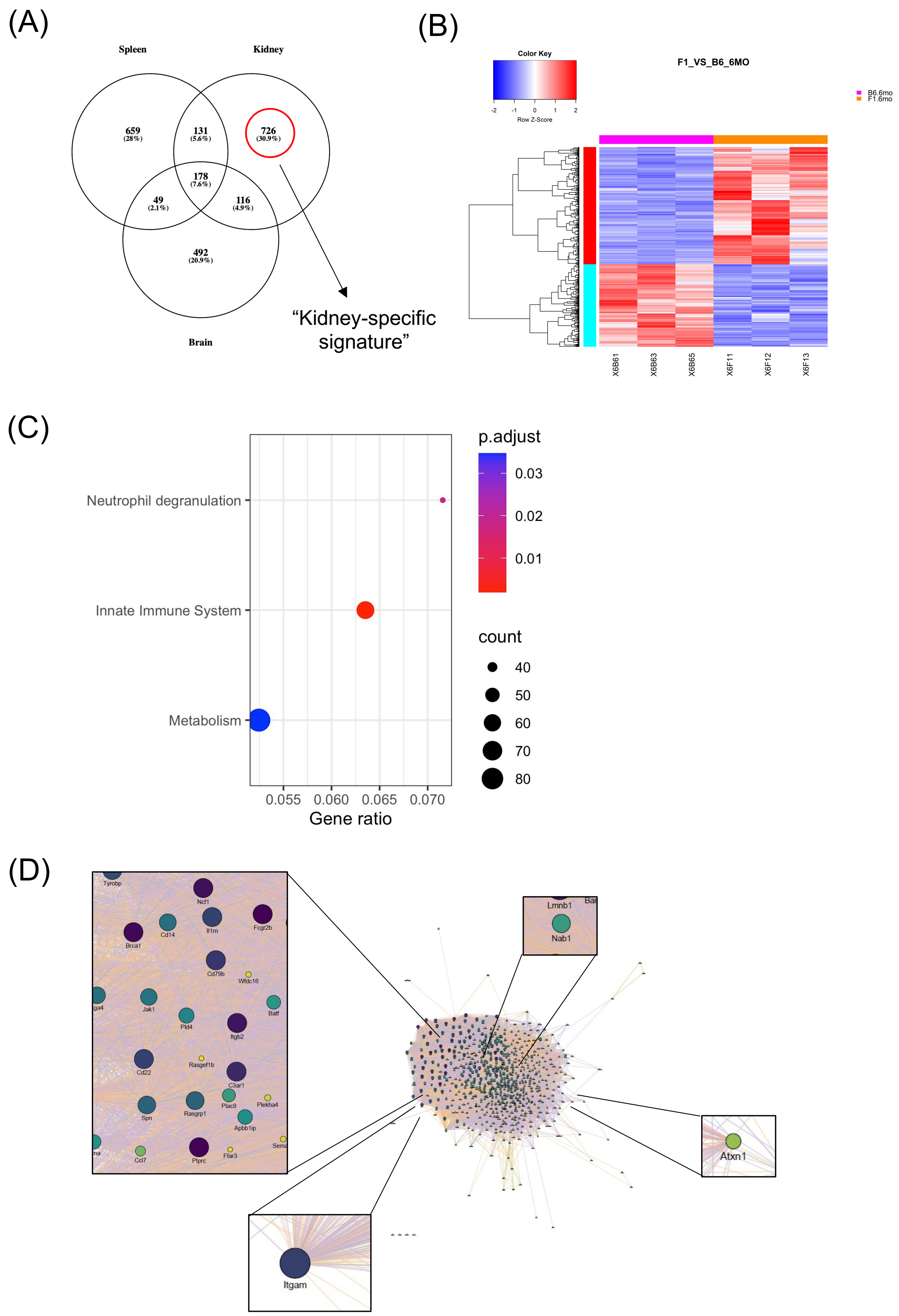

Frangou E, *et al. Ann Rheum Dis* 2022; 81:1409–1419. doi: 10.1136/annrheumdis-2021-222069

Fig. S1. Mouse kidney-specific transcriptome of nephritic lupus mice vs healthy mice. (**A**) Venn diagram demonstrating the comparison between differentially expressed genes (DEGs) within the spleen, kidneys, and brain from NZB/W-F1 lupus vs C57BL/6 healthy mice at the clinical (nephritic) stage of the SLE. The kidney-specific gene signature is defined by 726 genes that are differentially expressed only within kidneys but not in other tissues, (**B**) Heatmap of the 726 kidney-specific DEGs (425 upregulated, 301 downregulated), (**C**) Dot-plot diagram demonstrating functionally enriched REACTOME pathways of the 726 kidney-specific DEGs, (**D**) Gene-network representation of the 726 kidney-specific DEGs. Hub genes that correspond to lupus risk loci are depicted by larger size fonds.

Frangou E, *et al. Ann Rheum Dis* 2022; 81:1409–1419. doi: 10.1136/annrheumdis-2021-222069

Fig. S2

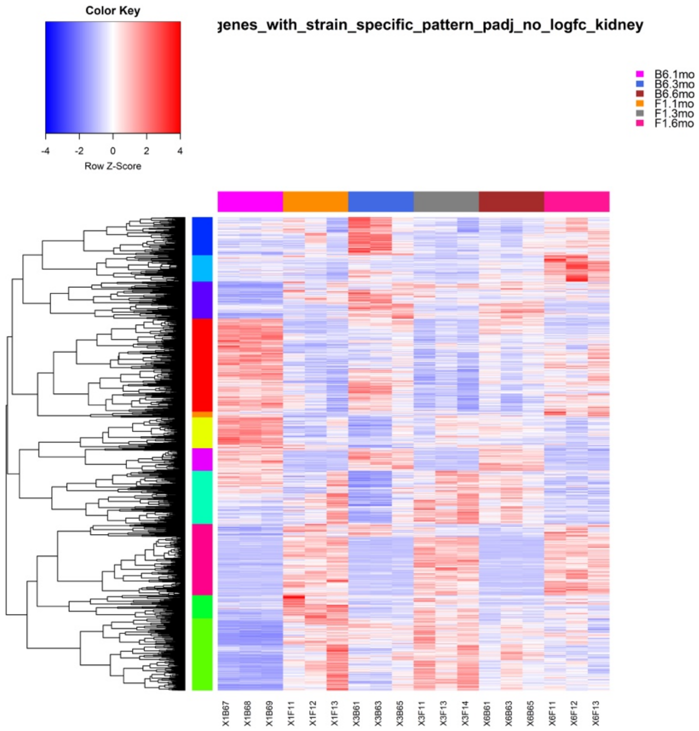

Frangou E, *et al. Ann Rheum Dis* 2022; 81:1409–1419. doi: 10.1136/annrheumdis-2021-222069

BMJ Publishing Group Limited (BMJ) disclaims all liability and responsibility arising from any reliance placed on this supplemental material which has been supplied by the author(s)

Fig. S2. Time-series analysis. Heatmap showing the differentially expressed genes (DEGs) demonstrating a strain-specific pattern within the kidneys from NZB/W-F1 lupus mice (F1) vs C57BL/6 healthy mice (B6) at the pre-puberty (1mo), pre-autoimmunity (3mo) and nephritic (6mo) stage of the disease.

Frangou E, *et al. Ann Rheum Dis* 2022; 81:1409–1419. doi: 10.1136/annrheumdis-2021-222069

Fig. S3

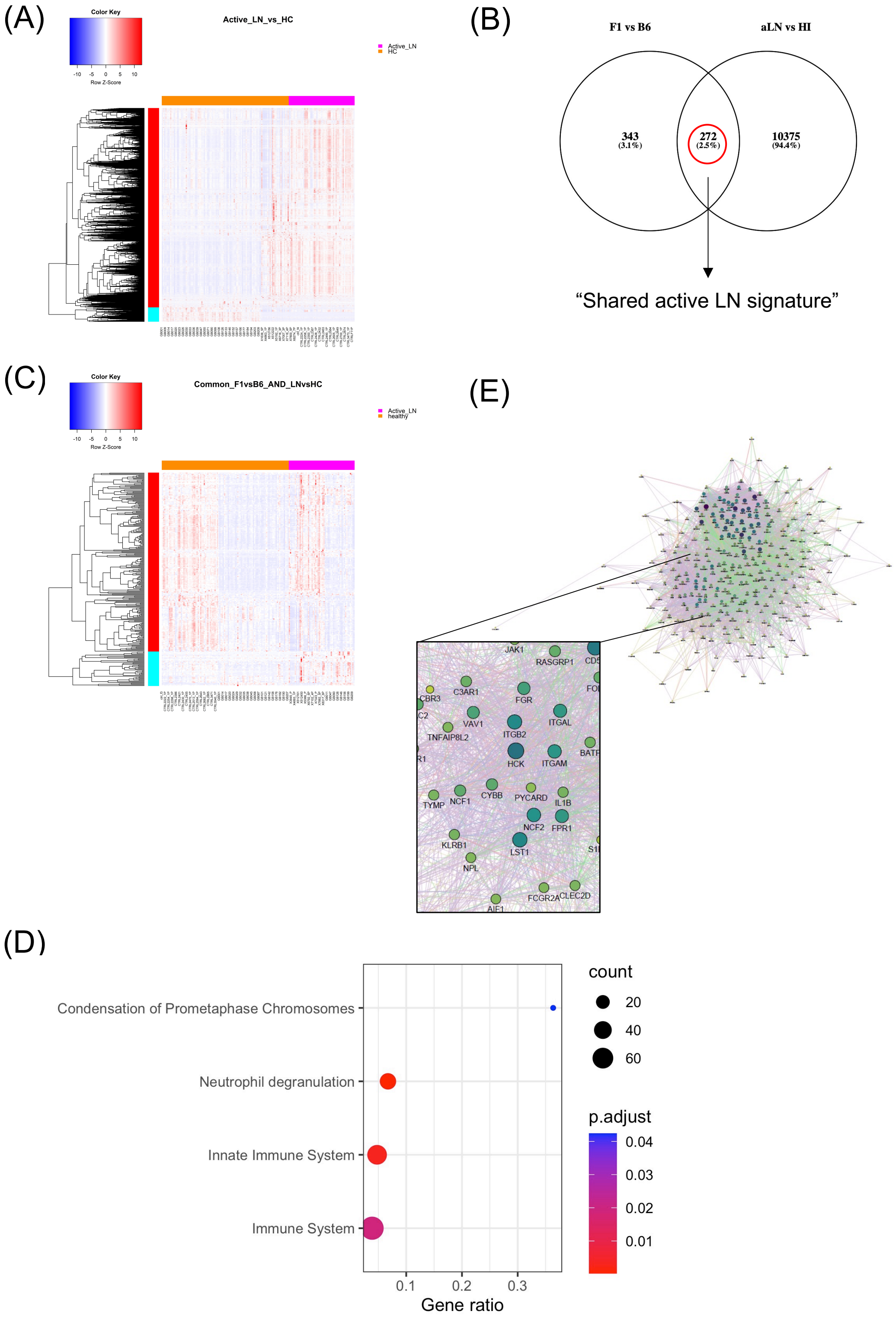
(A)
Color Key
Row Z-Score
Active_LN_vs_HC
Active_LN
HC
(B)
F1 vs B6
aLN vs HI
343
(3.1%)
272
(2.5%)
10375
(94.4%)
"Shared active LN signature"
(C)
Color Key
Row Z-Score
Common_F1vsB6_AND_LNvsHC
Active_LN
healthy
(E)
JAK1
RASGRP1
C3AR1
FGR
VAV1
ITGB2
ITGAL
TNFAIP8L2
HCK
ITGAM
TYMP
NCF1
CYBB
PYCARD
IL1B
NCF2
FPR1
KLRB1
LST1
NPL
FCGR2A
CLEC2D
AIF1
(D)
Condensation of Prometaphase Chromosomes
Neutrophil degranulation
Innate Immune System
Immune System
Gene ratio
count
20
40
60
p.adjust
0.04
0.03
0.02
0.01

Fig. S3. Common genes between the kidney-specific gene expression profile from lupus vs healthy mice at the clinical (nephritic) stage and the whole-blood gene expression profile from active LN (aLN) patients vs healthy individuals (HI) defines a "shared active LN signature". (**A**) Heatmap of the 10647 differentially expressed genes (DEGs) in the whole-blood from aLN patients vs HI, (**B**) Venn diagram demonstrating the comparison between the orthologous genes of the mouse kidney-specific DEGs from NZB/W-F1 lupus vs C57BL/6 healthy mice at the clinical (nephritic) stage and the whole-blood DEGs from aLN patients vs HI. The common "shared active LN signature" is defined by the union of the Venn diagram, corresponding to 272 common genes, (**C**) Heatmap of the "shared active LN signature", composed of 272 genes (193 upregulated, 79 downregulated), (**D**) Dot-plot diagram demonstrating functionally enriched REACTOME pathways of the "shared active LN signature", (**E**) Gene network representation of the "shared active LN signature". Hub genes that correspond to lupus risk loci are depicted by larger fond size.

Fig. S4

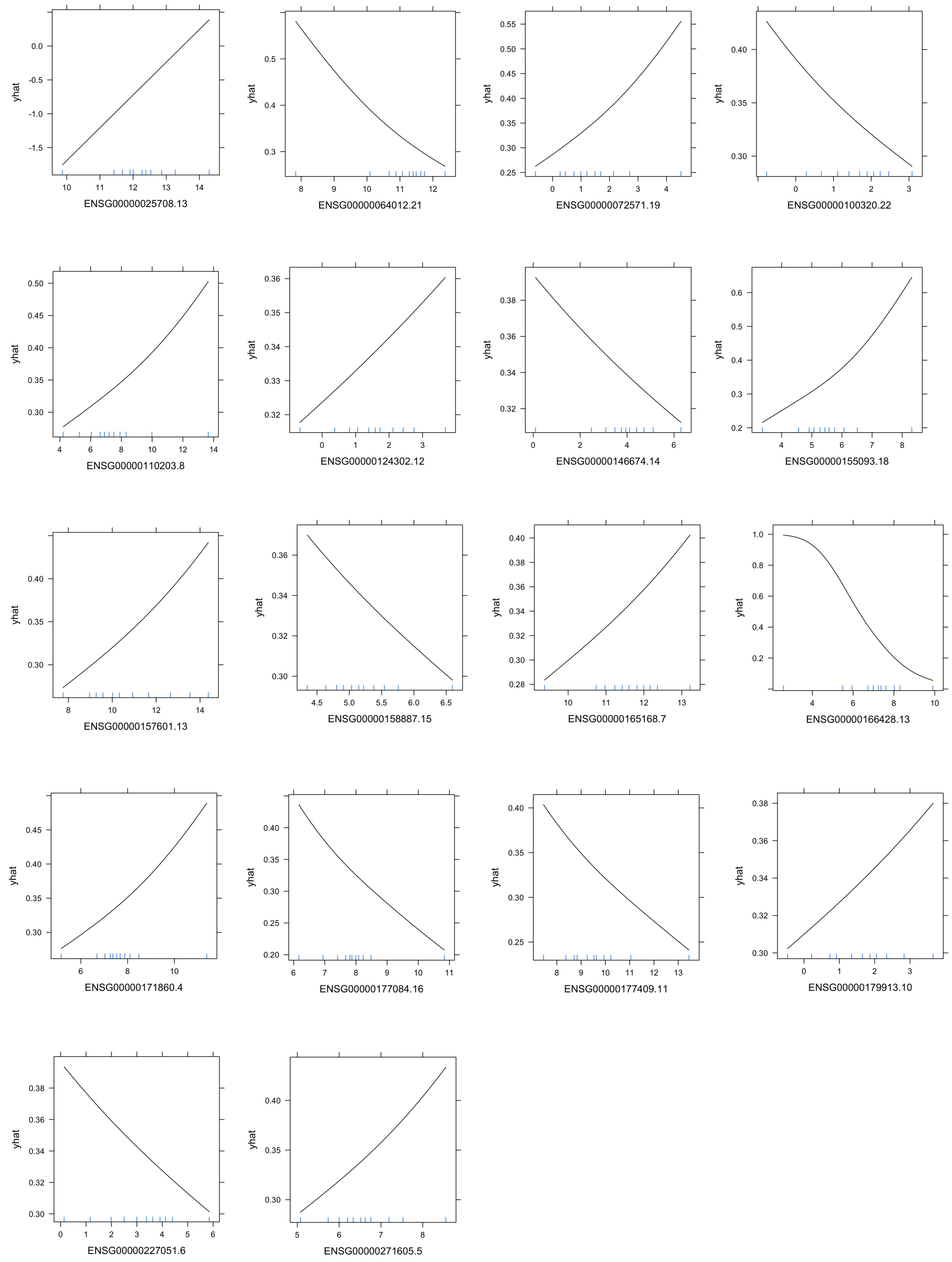

Frangou E, *et al*. *Ann Rheum Dis* 2022; 81:1409–1419. doi: 10.1136/annrheumdis-2021-222069

Fig. S4. Partial Dependence Plots (PDPs) of the probability of predicting patients with active LN compared to healthy individuals based on all model predictors. The x-axis represents the normalized gene expression value and y-axis is the probability of predicting active LN status.

Frangou E, *et al. Ann Rheum Dis* 2022; 81:1409–1419. doi: 10.1136/annrheumdis-2021-222069

Fig. S5

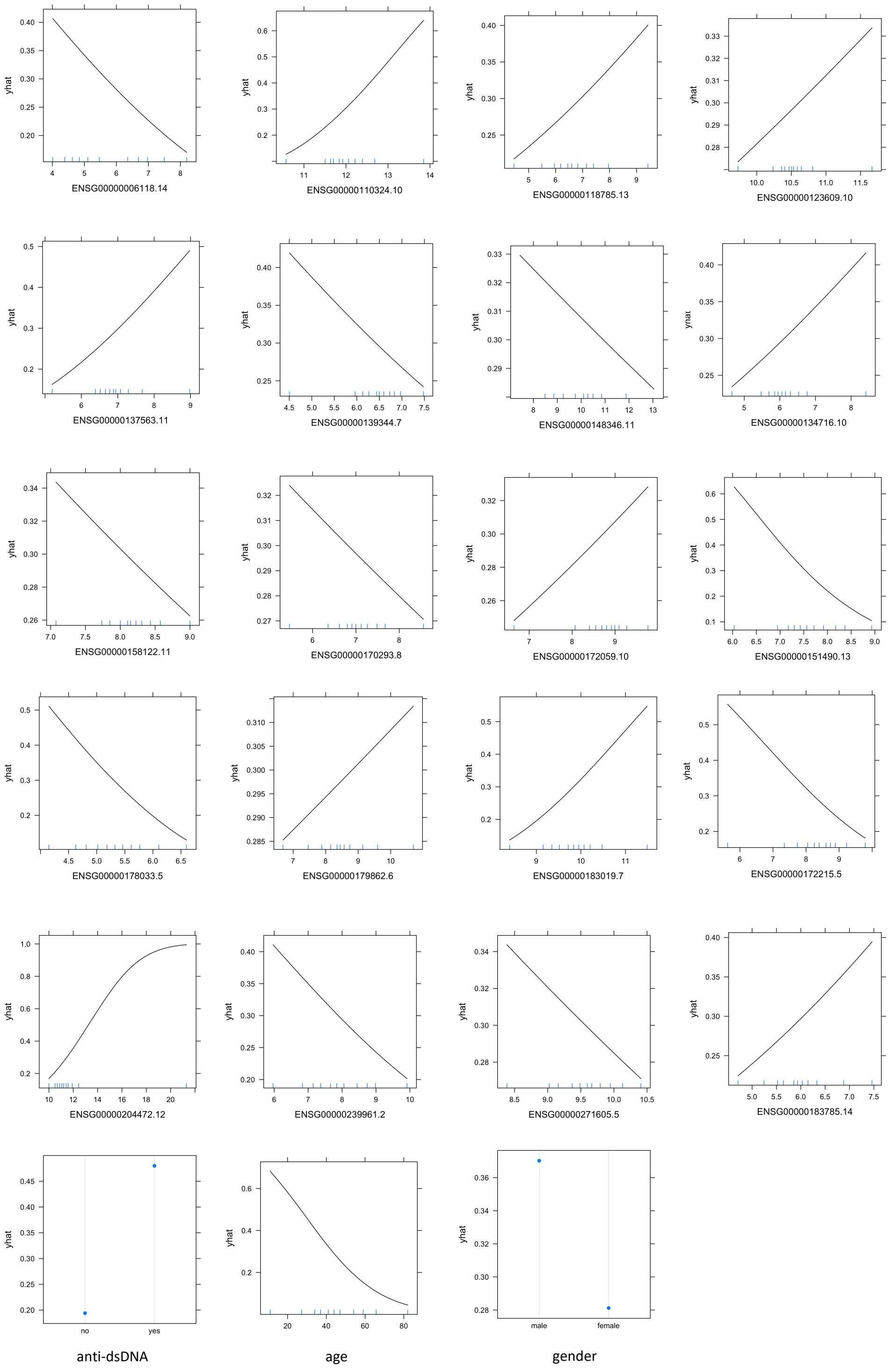
ENSG00000006118.14
ENSG00000110324.10
ENSG00000118785.13
ENSG00000123609.10
ENSG00000137563.11
ENSG00000139344.7
ENSG00000148346.11
ENSG00000134716.10
ENSG00000158122.11
ENSG00000170293.8
ENSG00000172059.10
ENSG00000151490.13
ENSG00000178033.5
ENSG00000179862.6
ENSG00000183019.7
ENSG00000172215.5
ENSG00000204472.12
ENSG00000239961.2
ENSG00000271605.5
ENSG00000183785.14
anti-dsDNA
age
gender

Fig. S5. Partial Dependence Plots (PDPs) of the probability of predicting active LN patients compared to non-LN patients based on all model predictors. The x-axis represents the normalized gene expression value or category for categorical variables and y-axis is the probability of predicting active LN status.

REFERENCES

1. Hochberg MC. Updating the American College of Rheumatology revised criteria for the classification of systemic lupus erythematosus. Arthritis and Rheumatism 1997;
2. Zen M, Iaccarino L, Gatto M et al. Lupus low disease activity state is associated with a decrease in damage progression in Caucasian patients with SLE, but overlaps with remission. Annals of the Rheumatic Diseases 2018;
3. Franklyn K, Lau CS, Navarra S V. et al. Definition and initial validation of a Lupus Low Disease Activity State (LLDAS). Annals of the Rheumatic Diseases 2016;
4. Nakou M, Knowlton N, Frank MB et al. Gene expression in systemic lupus erythematosus: Bone marrow analysis differentiates active from inactive disease and reveals apoptosis and granulopoiesis signatures. Arthritis and Rheumatism 2008; 58: 3541–3549.
5. Panousis NI, Bertsias GK, Ongen H et al. Combined genetic and transcriptome analysis of patients with SLE: distinct, targetable signatures for susceptibility and severity. Annals of the Rheumatic Diseases 2019;
6. Frangou E, Chrysanthopoulou A, Mitsios A et al. REDD1/autophagy pathway promotes thromboinflammation and fibrosis in human systemic lupus erythematosus (SLE) through NETs decorated with tissue factor (TF) and interleukin-17A (IL-17A). Annals of the Rheumatic Diseases 2019;
7. Theofilopoulos AN, Dixon FJ. Murine Models of Systemic Lupus Erythematosus. Advances in Immunology 1985;
8. Perry D, Sang A, Yin Y et al. Murine Models of Systemic Lupus Erythematosus. Journal of Biomedicine and Biotechnology *[Internet]* 2011; 2011: 1–19. Available from: http://www.hindawi.com/journals/bmri/2011/271694/
9. Lu L, Kaliyaperumal A, Boumpas D, Datta S. Major peptide autoepitopes for nucleosome-specific T cells of human lupus. J Clin Invest 199AD; 104: 345–355.
10. Andrews S. FastQC - A quality control tool for high throughput sequence data. http://www.bioinformatics.babraham.ac.uk/projects/fastqc/. Babraham Bioinformatics 2010;
11. Dobin A, Davis CA, Schlesinger F et al. STAR: Ultrafast universal RNA-seq aligner. Bioinformatics 2013;
12. Anders S, Pyl PT, Huber W. HTSeq-A Python framework to work with high-throughput sequencing data. Bioinformatics 2015;
13. Love MI, Huber W, Anders S. Moderated estimation of fold change and dispersion for RNA-seq data with DESeq2. Genome Biology 2014;
14. Robinson MD, McCarthy DJ, Smyth GK. edgeR: A Bioconductor package for differential expression analysis of digital gene expression data. Bioinformatics 2009;
15. Reimand J, Kull M, Peterson H, Hansen J, Vilo J. G:Profiler-a web-based toolset for functional profiling of gene lists from large-scale experiments. Nucleic Acids Research 2007;
16. Warde-Farley D, Donaldson SL, Comes O et al. The GeneMANIA prediction server: Biological network integration for gene prioritization and predicting gene function. Nucleic Acids Research 2010;
17. Chen EY, Xu H, Gordonov S, Lim MP, Perkins MH, Ma'ayan A. Expression2Kinases: mRNA profiling linked to multiple upstream regulatory layers. Bioinformatics 2012;
18. Zhao S, Guo Y, Sheng Q, Shyr Y. Heatmap3: An improved heatmap package with more powerful and convenient features. BMC Bioinformatics 2014;
19. Duan Q, Reid SP, Clark NR et al. L1000CDS2: LINCS L1000 characteristic direction

signatures search engine. npj Systems Biology and Applications 2016;

20. Kuhn M. Building predictive models in R using the caret package. Journal of Statistical Software 2008;
21. Robin X, Turck N, Hainard A et al. pROC: An open-source package for R and S+ to analyze and compare ROC curves. BMC Bioinformatics 2011;
22. Lê S, Josse J, Husson F. FactoMineR: An R package for multivariate analysis. Journal of Statistical Software 2008;
23. Greenwell BM. pdp: An R package for constructing partial dependence plots. R Journal 2017;
